# Assessing the Efficacy of Ultrasound Imaging for Diagnosing Appendicitis in Male Patients: A Retrospective Study

**DOI:** 10.7759/cureus.86749

**Published:** 2025-06-25

**Authors:** Waleed Khan, Ciara Mallon, Zahid Bahli, Cristina Croitoru, Andrew Carmichael, Rosin Kelly, Blaithnaid French, Tim White

**Affiliations:** 1 General Surgery, Altnagelvin Hospital, Londonderry, GBR

**Keywords:** appendicectomy, appendicitis, computed tomography, computerized tomography, sensitivity, ultrasound

## Abstract

Background: Inflammation in the vermiform region of the appendix is referred to as appendicitis. It can be diagnosed using clinical assessment and various imaging modalities. Among other imaging modalities, ultrasound (US) is an easily interpretable, cost-effective, and portable method. Unfortunately, the diagnosis of appendicitis presents a challenge due to its non-specific clinical presentation. In this respect, this study aimed to evaluate US sensitivity and specificity in diagnosing appendicitis in male patients.

Methods: This study was conducted at the Altnagelvin Hospital, Londonderry. We examined retrospective data on 237 male patients aged 14 years and above who presented with appendicitis and underwent US of the abdomen and/or appendix. We identified patients with confirmed appendicitis and/or secondary signs on imaging and those who underwent appendectomy with established inflammation at histology.

Results: In our study, the majority of patients were between the ages of 21 and 40 years (57.8%), with a smaller proportion of patients under 20 years (31.3%), between 41 and 60 years (7.0%), and over 60 years (3.9%). Interestingly, 9.3% of patients underwent appendectomy despite not having US-revealed appendicitis and the absence of secondary signs, while 24% of patients with no US-revealed appendicitis but with the presence of secondary signs underwent appendectomy, with 12% having pathological evidence of appendicitis. The sensitivity and specificity of ultrasound for diagnosing appendicitis were 81% and 96%, respectively. The proportion of US-revealed appendicitis was 20.5%, whereas other modalities (OM) showed positive results in 8.73% of cases. No association was observed between US-revealed appendicitis and comorbidities (p=0.99), whereas a significant association (p<0.01) was observed between appendicitis revealed by OM-based investigations (in particular, CT) and comorbidities.

Conclusion: US imaging could be a useful tool for diagnosing appendicitis, particularly in male patients; also, it can prevent unnecessary surgical intervention. Our findings suggest that US is an effective choice of imaging for initial diagnosis. However, in the case of associated pathologies, CT is the best choice for further evaluation.

## Introduction

Appendicitis refers to inflammation in the vermiform region of the appendix [1]. In most cases, lower abdominal symptoms in suspected patients are attributed to underlying appendicitis, which poses a reported life-threatening risk estimate of 8.6% in men compared to 6.7% in women [2]. Moreover, there is a slight male predominance among patients presenting before the age of 30, with a male-to-female ratio of approximately 3:2 [1]. The reported incidence rate of appendicitis in adults is around 100-150 per 100,000 person-years [2], while the incidence of acute appendicitis globally is reported to be 11 per 10,000 population per year. The overall risk and prevalence in the general population are estimated at 7%. Geographical differences have been reported, revealing that the lifetime risk of developing appendicitis in the USA is 9%, in Europe, 8%, and in Africa, 2%, based on the reported prevalence and risk factors. The occurrence of acute appendicitis has been reported across all age groups; however, it rarely develops at extreme ages, and most cases are reported to occur in the age group of 10-14 years in female patients, and 15-19 years in male patients [1].

Surgical removal of the appendix, known as appendectomy, has been considered the standard treatment. However, the presence of comorbidities raises concerns about the diagnosis and treatment of acute appendicitis. Antibiotic treatment is increasingly being considered a better option as a non-operative management strategy for uncomplicated appendicitis [3]. Despite these therapeutic options, several cases go untreated due to the high frequency of misdiagnosed cases [2]. The diagnosis of appendicitis presents a challenge due to its non-specific clinical presentation, which may overlap with a range of differential diagnoses, including non-specific abdominal pain, suspected appendicitis, and other differential diagnoses, such as irritable bowel syndrome (IBS), peptic ulcer disease (PUD), pancreatitis, inflammatory bowel disease (IBD), small bowel obstruction (SBO) or large bowel obstruction (LBO), abdominal aortic aneurysm (AAA), diabetic ketoacidosis, gastroenteritis, and others, thus creating diagnostic ambiguity [3]. In 2010, a study reported 69.2% misdiagnosed cases, with males comprising 57.6% of the total participants. For male adult appendicitis, diagnostic error rates range from 5.9% to 25.7% [4].

The misdiagnosis rate for acute appendicitis is approximately 9% in men and 23.2% in women. This discrepancy may be due to the more complex anatomy on the right side of a woman's abdomen, including the ovaries, uterus, and fallopian tubes. Moreover, the issue could arise from an infection of the ovary or uterus, or ectopic pregnancy, where a fertilized ovum implants outside the uterus. Conversely, in men, an inflamed lymph node or a viral infection of the intestinal tract could be the underlying cause [5]. The clinical diagnosis of acute appendicitis relies on the patient's medical history, physical examination, and laboratory tests. Diagnosis is usually straightforward when patients present with typical signs and symptoms, while atypical presentation may lead to diagnostic confusion and a delay in treatment. When clinical diagnosis is uncertain, imaging modalities such as ultrasound (US), computed tomography (CT), and magnetic resonance imaging (MRI) can be considered as further means of investigation [6].

The use of imaging modalities to diagnose acute appendicitis enhances the sensitivity and specificity of the diagnostic process. Nevertheless, a critical issue is establishing accurate diagnostic criteria that can effectively differentiate uncomplicated from complicated cases of acute appendicitis, given the variable diagnostic accuracy of imaging techniques [7]. Among other imaging modalities, US has been widely used as a technique for diagnosing suspected appendicitis and as a basis for further clinical examination. In this regard, the current study aimed to evaluate the accuracy of ultrasound in diagnosing acute appendicitis in male patients compared to other imaging modalities such as CT and MRI. Moreover, we endeavored to identify the sensitivity and specificity of US in diagnosing appendicitis in male patients and to provide recommendations for the clinical application of US in diagnosing acute appendicitis in male patients based on the study findings.

## Materials and methods

​​​​Methodology

A retrospective cross-sectional descriptive research study was undertaken at the Altnagelvin Hospital, Londonderry, between 2015 and 2022, targeting male patients due to their less complicated reproductive anatomy. The study aimed to evaluate the efficacy of ultrasound imaging, compared with other imaging modalities such as CT, CT arterial portography (CTAP), CT of the kidney, ureters and bladder (CTKUB), and oesophago-gastro-duodenoscopy CT (OGD-CT), in the diagnosis of appendicitis. The reports from these imaging modalities were gathered for the assessment of appendicitis. The study employed a non-probability sampling technique to collect clinical reports of patients from the hospital database.

Selection Criteria

The study focused on male patients aged 14 years and above who presented with suspected appendicitis and underwent abdominal ultrasound and/or dedicated ultrasound of the appendix. Patients with confirmed appendicitis and/or secondary signs on imaging and those who underwent appendicectomy with confirmed inflammation at histology were identified. Female patients with suspected appendicitis who underwent abdominal ultrasound and/or dedicated ultrasound of the appendix were excluded from the study due to variations in anatomy.

Statistical Analysis

The Shapiro-Wilk test was conducted to determine the normality of the data. Descriptive analysis was performed for qualitative and categorical variables at a nominal scale, including age, CRP levels, referral source, visualization of the appendix on ultrasound and other modalities (OM), and associated pathologies and reports of appendectomy performed in patients. The demographic data were presented in frequencies and percentages. The chi-square test was independently employed to determine the comparative association between US imaging or other modalities and associated pathologies and referrals from different departments. Additionally, the results of US were correlated with appendectomy. Various parameters such as accuracy, sensitivity, specificity, positive predictive value (PPV), negative predictive value (NPV), and disease prevalence were expressed as percentages. The confidence intervals (CIs) for accuracy, sensitivity, and specificity were reported using the "exact" Clopper-Pearson CI. A p-value less than 0.05 was considered statistically significant.

Ethical Approval

This research study was conducted per ethical principles and was approved by the Altnagelvin Hospital, Londonderry. The participants' confidentiality and anonymity were maintained and no form of incentive or compensation was provided to the participants.

## Results

Demographic features

**Figure 1:**
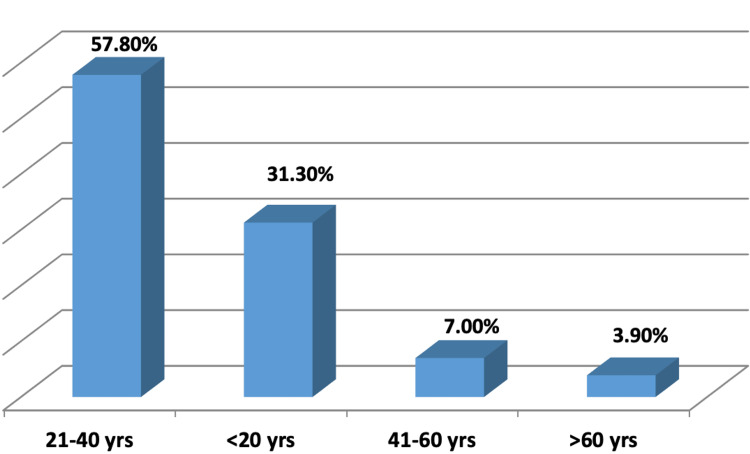
Age distribution of patients Mean ± SD : 29.12 ± 14.11 years

Records for appendicitis for a total of 237 patients were extracted from the local hospital database. From these, eight patients were excluded from the study: 5 patients (2.10%) were not assessed for their appendix, 1 patient (0.42%) had a history of appendectomy, 1 patient (0.42%) had only a CTAP report, and 1 patient (0.42%) had no US report. The remaining 229 patients were included in the analysis, out of which 62 patients underwent appendicectomy, and 43 of the 229 patients (18.7%) were pathologically confirmed for appendicitis. The age distribution of patients was as follows: 72 patients (31.3%) were under 20 years old, 133 patients (57.8%) were in the 21-40 year age group, 16 patients (7.0%) were in the 41-60 year age group, and 9 patients (3.9%) were over 60 years old. Patients' overall mean age (± standard deviation) was 29.12 (± 14.11) years (Figure 1).

Appendicitis with positive US visualization

Out of the 237 patients identified, 50 patients were diagnosed with acute appendicitis based on US reports of appendicitis. Among these patients, 40 out of 50 (80%) underwent appendicectomy; 15 (30%) were treated conservatively and discharged after improvement. Only one patient out of the 50 (2%) received a CT scan, which confirmed the presence of appendicitis, but did not undergo appendicectomy. Furthermore, the laboratory parameter of CRP levels was assessed in these patients. The mean CRP level for patients with visualized appendicitis was 41 mg/L (normal value <5), while those who underwent appendicectomy had a mean CRP level of 31 mg/L. Among those who underwent appendicectomy and had pathological evidence, the mean CRP level was 35 mg/L (Table 1).

**Table 1:** Diagnosis of appendicitis based on ultrasound (US) visualization and secondary signs

	Appendicitis with positive US visualization	Appendicitis with negative US visualization	Appendicitis with negative US visualization and positive secondary signs
Frequency	50	149	25
Secondary signs	Absent	Absent	Present
Appendectomy	40 (80%)	14 (9.39%)	6 (24%)
Other treatment	15 (30%)	0	0
Appendicitis with no appendectomy	1 (2%)	0	3 (12.5%)
Appendectomy with a normal appendix	0	2 (1.34%)	0
CT scan following ultrasound	1 (2%)	0	18 (72%)
Mean CRP for visualized appendicitis (mg/L)	41	41	0
Mean CRP for appendectomy (mg/L)	31	45	0
Mean CRP for appendectomy with pathology (mg/L)	35	0	0

Appendicitis with negative US visualization and negative secondary signs

Of 237 patients, 149 did not show appendicitis in US, and no secondary signs (free fluid, ileus, fat stranding, abscess, lymphadenopathy, bowel wall edema, abnormal adjacent bowel, and appendicoliths) of the condition were observed. Among these patients, 14 out of 149 (9.39%) underwent appendicectomy. Conversely, 2 out of 149 patients (1.34%) underwent appendicectomy with confirmed acute appendiceal pathology, despite having negative US findings and no evidence of secondary signs. Among these, a mean CRP of 45 mg/L for patients who underwent appendicectomy and a mean CRP of 41 mg/L for patients with positive appendicitis pathology were observed (Table 1).

Appendicitis with negative US visualization and positive secondary signs

A total of 25 patients had negative visualization for appendicitis in US, but they were found to have positive findings of secondary or other signs. Of these, 6 out of 25 (24%) patients underwent appendectomy, while 3 out of 25 (12%) had pathological evidence of appendicitis. Additionally, 18 out of 25 patients underwent a CT scan in addition to their ultrasound examination (Table 1), with 4 patients proven to have appendicitis on the scan.

Appendicitis with positive US visualization on US and CT scans

Among the 229 patients, only 1 (0.4%) had a visualized appendix on ultrasound and CT that led to appendicectomy with a positive pathological report. Another patient with visualized appendicitis on ultrasound and CT (0.4%) was treated conservatively. However, 7 patients (3.05%) underwent a CT scan despite not having a visualized appendix with or without secondary signs, and no patients were identified with appendicitis following CT.

Appendicitis and other modalities

Association of Appendicitis With Positive and Negative Visualization

**Figure 2:**
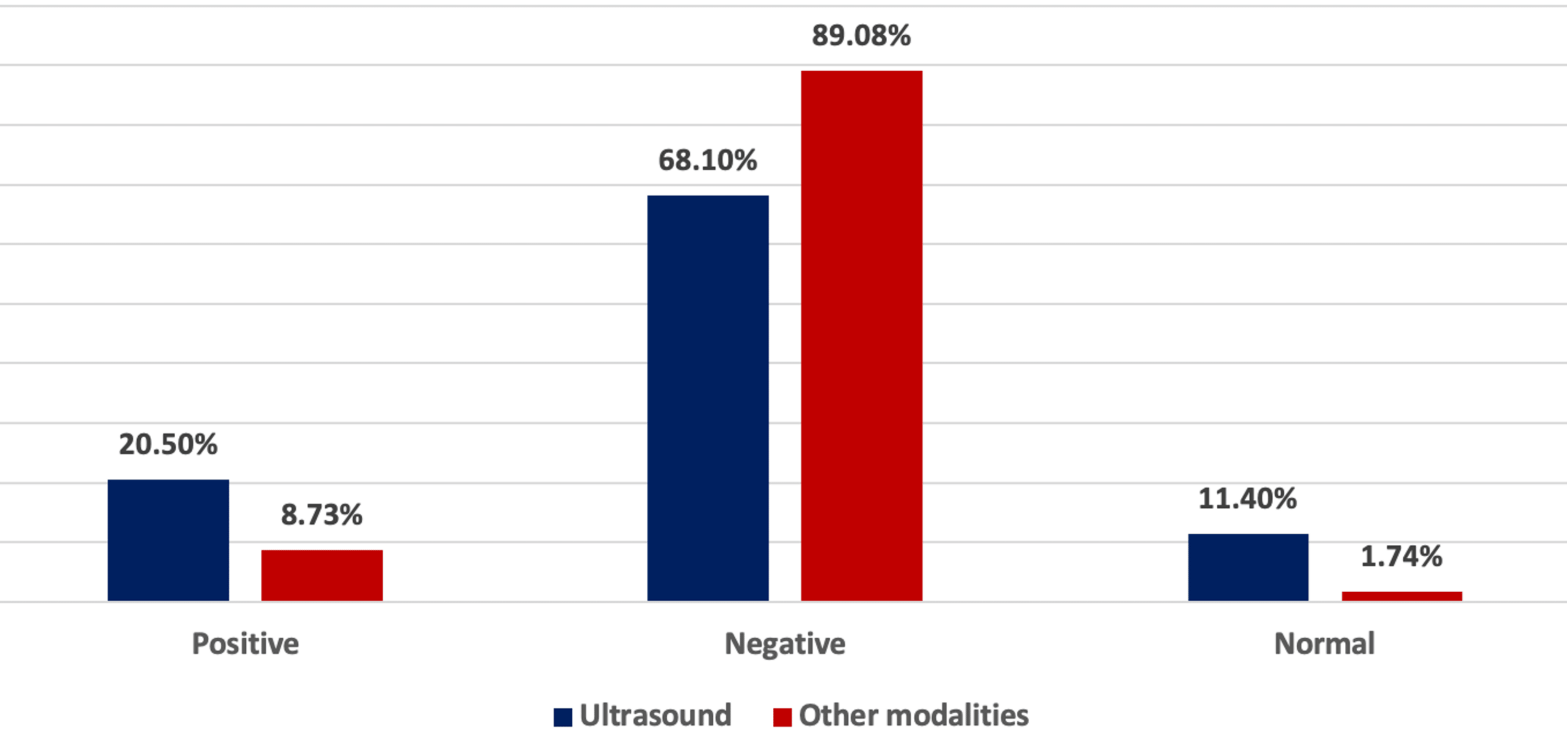
Prevalence of appendicitis based on ultrasound imaging and other modalities

A significant association was observed between the presence of appendicitis in patients who underwent appendectomy and those with documented visualization of appendicitis on ultrasound, as evidenced by a p-value less than 0.001 (Table 2). Ultrasound detected appendicitis in 20.5% of cases, while other modalities such as CT, CTAP, CTKUB, and OGD showed positive results in 8.73% of cases (Figure 2), with CT accounting for 25%, CTAP for 20%, CTKUB 10%, and OGD 5%. While there was no statistically significant association between the presence of appendicitis on ultrasound and associated pathologies (Table 3), the identification of appendicitis by other modalities and related pathologies showed a significant association (p<0.001) (Table 4).

**Table 2:** Association of appendectomies with ultrasound (US)-visualized appendicitis

Appendectomy	US-visualized appendicitis				
	Acute appendicitis, N (%)	Appendicitis, N (%)	Appendicitis not visualized, N (%)	Appendix visualized - normal, N (%)	p-value
Yes	22 (84.6)	12 (57.1)	22 (14.1)	6 (23.1)	<0.001
No	4 (15.4)	9 (42.9)	134 (85.9)	20 (76.9)	

**Table 3:** Association between ultrasound (US)-visualized appendicitis and comorbidities p-value, 0.999 (no significant association)

Comorbidities	US-visualized appendicitis, N (%)			
	Acute appendicitis	Appendicitis	Appendicitis not visualized	Appendix (normal)
No pathology	24 (92.3)	18 (85.7)	105 (67.3)	20 (76.9)
Hepatobiliary inflammation	-	1 (4.8)	-	-
Lymph nodes	-	-	4 (2.6)	-
Splenomegaly	1 (3.8)	-	1 (0.6)	-
Inflammation of the caecum	-	-	1 (0.6)	-
Cholelithiasis and fatty liver	-	-	1 (0.6)	-
Complex inflammatory collection in the abdomen	-	-	1 (0.6)	-
Debris in the gallbladder and bladder	1 (3.8)	-	-	-
Enteritis vs. inflammatory bowel disease	-	-	1 (0.6)	-
Fatty liver with a dilated common bile duct and abdominal aortic aneurysm	-	1 (4.8)	5 (3.2)	1(3.8)
Fatty liver with renal cyst and liver debris	-	-	1 (0.6)	-
Crohn's flares with thickened small bowel	-	-	1 (0.6)	-
Free fluid in the right iliac fossa (RIF), adenitis	-	-	3 (1.9)	-
Gallbladder polyps	-	-	4 (2.6)	-
Gastritis	-	-	1 (0.6)	-
Dilated gallbladder with sludge	-	-	1 (0.6)	-
Hepatic cyst	-	-	1 (0.6)	-
Ileocolic lymph nodes	-	-	1 (0.6)	-
Incidental urachal cyst	-	-	1(0.6)	-
Intussusception on ultrasound	-	-	-	1(3.8)
Leak from the caecum and multifocal abscess formation	-	-	1 (0.6)	-
Mesenteric adenitis	-	-	5 (3.2)	1(3.8)
Duodenal ulcer	-	-	1 (0.6)	-
Renal cyst	-	-	2 (1.3)	-
Right-sided colitis	-	-	1 (0.6)	-
Gallbladder stone and renal stone	-	-	3 (1.9)	-
Psoas muscle hematoma	-	-	1 (0.6)	-
Short bowel loop in the RIF (resembles the appendix)	-	-	1 (0.6)	-
Ureterocele, liver hemangioma	-	-	-	1(3.8)
Sigmoid diverticulitis	-	-	1 (0.6)	-
Terminal ileal fatty infiltration	-	-	1 (0.6)	-
Cyst in the prostate gland	-	-	1 (0.6)	-
Echogenic foci in the liver	-	-	-	1(3.8)
Focal abnormality in the liver	-	-	1 (0.6)	-
Multiple hemangiomas with gallbladder polyps	-	-	1 (0.6)	-
Subcentric hepatic hemangioma with hepatic cyst	-	-	1 (0.6)	-
Small abdomen and groin lymph nodes with splenomegaly	-	-	1 (0.6)	-
Subcentric lymph nodes in the RIF	-	-	1 (0.6)	1 (3.8)

**Table 4:** Association of OM-visualized appendicitis and comorbidities OM: other modalities; CT: computed tomography; CTAP: CT arterial portography; CTKUB: CT of the kidney, ureters and bladder; OGD: oesophago-gastro-duodenoscopy p-value, <0.001 (significant association)

Comorbidities	OM-visualized appendicitis, N (%)			
	CT	CTAP	CTKUB	OGD
No pathology	8 (53.3)	2 (50)	2 (100)	-
Hepatobiliary inflammation	-	-	-	-
Lymph nodes	1 (6.7)	-	-	-
Splenomegaly	-	-	-	-
Inflammation of the caecum	-	-	-	-
Cholelithiasis and fatty liver	-	-	-	-
Complex inflammatory collection in the abdomen	1 (6.7)	-	-	-
Debris in the gallbladder and bladder	-	-	-	-
Enteritis vs. inflammatory bowel disease	-	-	-	-
Fatty liver with a dilated common bile duct and abdominal aortic aneurysm	2 (13.3)	-	-	-
Fatty liver with renal cyst and liver debris	-	-	-	-
Crohn's flares with thickened small bowel	-	-	-	-
Free fluid in the right iliac fossa (RIF), adenitis	-	-	-	-
Gallbladder polyps	-	-	-	-
Gastritis	-	-	-	1 (100)
Dilated gallbladder with sludge	-	-	-	-
Hepatic cyst	-	-	-	-
Ileocolic lymph nodes	-	-	-	-
Incidental urachal cyst	-	-	-	-
Intussusception on ultrasound	1 (6.7)	-	-	-
Leak from the caecum and multifocal abscess formation	1 (6.7)	-	-	-
Mesenteric adenitis	-	-	-	-
Duodenal ulcer	-	1 (25)	-	-
Renal cyst	-	-	-	-
Right-sided colitis	-	1 (25)	-	-
Gallbladder stone and renal stone	1 (6.7)	-	-	-
Psoas muscle hematoma	-	-	-	-
Short bowel loop in the RIF (resembles the appendix)	-	-	-	-
Ureterocele, liver hemangioma	-	-	-	-
Sigmoid diverticulitis	-	-	-	-
Terminal ileal fatty infiltration	-	-	-	-
Cyst in the prostate gland	-	-	-	-
Echogenic foci in the liver	-	-	-	-
Focal abnormality in the liver	-	-	-	-
Multiple hemangiomas with gallbladder polyps	-	-	-	-
Subcentric hepatic hemangioma with a hepatic cyst	-	-	-	-
Small abdomen and groin lymph nodes with splenomegaly	-	-	-	-
Subcentric lymph nodes in the RIF	-	-	-	-

**Figure 3:**
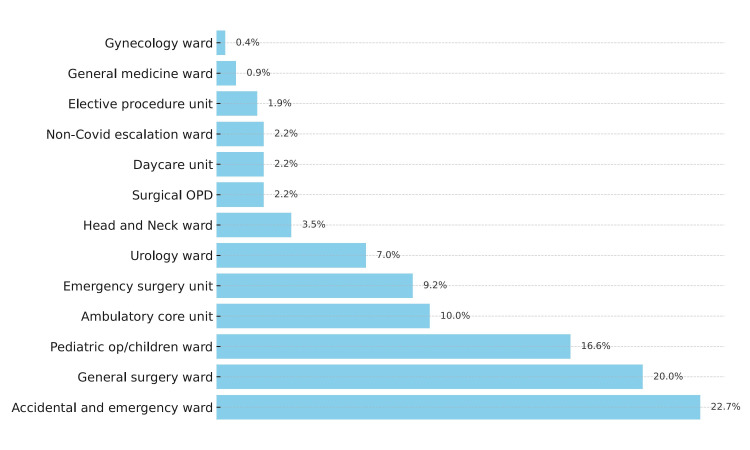
Frequency of reference sources for appendicitis visualization

Patients with suspected appendicitis were referred for further image-based evaluation by either the General Surgery department or the Accident and Emergency department (Figure 3). However, no significant association was observed between the departments that referred cases for visualization of appendicitis on ultrasound and other modalities (Table 5). Furthermore, the diagnostic accuracy of ultrasound imaging in confirmed cases of appendicitis displayed a sensitivity of 81.4% and a specificity of 96.43% at a 95% CI (Table 6).

**Table 5:** Association between the sources of referral for appendicitis visualization on ultrasound (US) and other modalities (OM) CT: computed tomography; CTAP: CT arterial portography; CTKUB: CT of the kidney, ureters and bladder; OGD: oesophago-gastro-duodenoscopy; op: outpatient

Reference source	US, N (%)				p-value	OM, N (%)				p-value
	Acute appendicitis	Appendicitis	Appendix not visualized	Appendix visualized (normal)		CT	CTAP	CTKUB	OGD	
Accident and emergency ward	3 (5.8)	7 (13.5)	39 (75)	3 (5.8)	0.572	4 (7.7)	2 (3.8)	1 (1.9)	-	0.994
Ambulatory core unit	-	-	18 (78.3)	5 (21.7)		5 (21.7)	-	-	-	
Pediatric op/children's ward	9 (23.7)	4 (10.5)	21 (55.3)	4 (10.5)		-	-	1 (2.6)	-	
GP	-	-	1 (100)	-		-	-	-	-	
Elective op	-	-	-	1 (100)		-	-	-	-	
Elective procedure unit	-	-	3 (100)	-		1 (33.3)	-	-	-	
Emergency assessment unit	-	-	1 (100)	-		-	-	-	-	
Emergency surgery unit	3 (14.3)	2 (9.5)	13 (61.9)	3 (14.3)		1 (4.8)	-	-	-	
Surgical OPD	0	2 (40)	2 (40)	1 (20)		-	-	-	-	
Urology ward	1 (6.3)	1 (6.3)	12 (75)	2 (12.5)		1 (6.3)	-	-	1 (6.3)	
Daycare unit	-	-	4 (80)	1 (20)		-	-	-	-	
Non-COVID escalation ward	1 (20)	1 (20)	3 (60)	-		-	-	-	-	
General surgery ward	8 (17.4)	3 (6.5)	30 (65.2)	5 (10.9)		3 (6.5)	2 (4.3)	-	-	
General medicine ward	-	-	1 (50)	1 (50)		-	-	-	-	
Gynecology ward	-	-	1 (100)	-		-	-	-	-	
Head and neck ward	1 (12.5)	1 (12.5)	6 (75)	-		-	-	-	-	

**Table 6:** Measurement of ultrasound performance for determining appendicitis

Sensitivity pattern on ultrasound	Values (CI), %
Sensitivity	81.4%
Specificity	96.43%
Positive likelihood ratio	22.79%
Negative likelihood ratio	0.19294
Positive predictive value	85.07%
Negative predictive value	95.5%
Accuracy	92.9%

## Discussion

Acute appendicitis, characterized by inflammation of the appendix, is the most frequent cause of abdominal pain, with a reported prevalence rate of 7%. However, its diagnosis remains challenging despite recent advancements, and selecting an appropriate imaging modality is crucial. Ultrasound is the preferred diagnostic imaging modality and is often used following clinical evaluations in patients suspected of having acute appendicitis. US imaging is the most convenient and viable option for children, whereas in adults, the results are often ambiguous. Several studies have shown that US is the most appropriate imaging technique for both obese and non-obese patients, owing to the high proportion of extra-mesenteric fat present [8].

In contrast, our study found that 9.3% of patients underwent appendectomy despite the absence of US-detected appendicitis and secondary signs of appendicitis. This finding is consistent with the observations made by Cuschieri et al. in 2008, who reported that 8.1% of patients who underwent urgent appendectomy had negative ultrasound imaging results [9]. Consistently, Sammalkorpi et al. in 2017 reported a negative appendicitis rate (NAR) at 8.7% of US-visualized appendicitis [10]. In a similar vein, Maloney et al. in 2019 reported that among 1435 appendectomies, the rate of US-revealed pathological appendicitis was 91.1% (1307/1435) with an NAR of 8.9% (128/1435) [11]. Another study manifested inconclusive US results in 39.4% of cases, with 29% of these patients ultimately diagnosed with appendicitis and an overall NAR of 5.9%. In contrast, Benedetto et al. in 2019 evaluated 139 patients suspected of acute appendicitis using ultrasound imaging and reported a prevalence of 50.35% for acute appendicitis with an NAR of 0% [10,12]. Disparities in diagnosis can be influenced by numerous factors, including inter-observer variability, differences in patient populations (such as age, body habitus, and coexisting medical conditions), and imaging timing that may also play a crucial role in diagnostic accuracy. Early imaging in acute appendicitis may yield lower sensitivity rates, as the inflammation may not have progressed to the point where it can be detected by imaging [13].

In cases where no appendicitis was detected on US imaging but secondary signs were present, a significant (statistical) proportion of patients (24%) underwent appendectomy, and a subset of these patients (12%) were found to have pathological evidence of appendicitis. The absence of a US-visualized appendix in the presence of secondary signs may be attributed to an incomplete investigation of the appendix, leading to failure in identifying certain types of appendicitis, such as segmental or tip appendicitis [14]. Additionally, overestimating the appendiceal diameter may lead to a false positive diagnosis [15]. Anatomical variations, such as retro-caecal appendix location, can further complicate the US diagnosis, as it can be technically difficult to visualize the appendix due to the overlying bowel gas/fecal artifact [16,17]. These findings underscore the need to carefully interpret ultrasound results, considering the imaging modality's limitations and potential confounding factors.

In our study, the prevalence of US-revealed appendicitis was 20.5%, whereas other imaging modalities were positive in 8.73% of cases. A recent investigation reported a lower incidence of US-revealed appendicitis (6.3%) and a higher incidence of CT-revealed appendicitis (22.5%) [1]. However, in another study, the overall prevalence of US-revealed appendicitis was 35.9%; CT was performed in 50.3% and CT after US in 13.8% of cases [18]. Moreover, a study by Sammalkorpi et al. in 2017 demonstrated that US and CT imaging had comparable accuracy rates of 78.8% [10]. These variations in efficacy among imaging modalities may be influenced by the observer, diagnostic approach, and imaging modality utilized [19].

However, CT has its advantages, with 100% reported sensitivity and the ability to perform the study in a way that is much less operator dependent; also, it is preferred in cases where ultrasound is difficult to perform, such as obese patients. Despite the advantages of CT compared to US for the diagnosis of appendicitis, studies demonstrate the excellent positive predictive value of US; if the appendix is visualized and is identified as abnormal, it is an indicator for surgery. However, in patients with negative visualization on US but the presence of secondary signs, a CT examination is recommended. This approach has clearly been shown to be cost-effective and safe for children and adults [8].

Our study found no significant (statistical) association between the detection of appendicitis on US and comorbidities. However, there was a significant association between appendicitis detected by OM (in particular, CT) and comorbidities. Similarly, Pelin et al. showed that a greater proportion of patients with acute appendicitis complicated with comorbidities were principally diagnosed by CT than with US, with a significant association seen (p-value 0.012) [20]. The CT-based identification of comorbidities evinces its high accuracy, sensitivity and specificity, and good visualization of the anatomy. Interestingly, we also observed that patients with a normal appendix on ultrasound often had other associated pathologies, such as hepatobiliary inflammation, fatty liver, abdominal aortic aneurysm, and debris in the gallbladder and urinary bladder. Emre et al. conducted a retrospective study reporting that among 1255 appendectomy recipients, 94% had appendicitis, with the majority of cases associated with phlegmonous appendicitis and gangrenous appendicitis with perforation [21]. Considering these reports, it can be established that a CT scan should be performed in the case of negative US imaging and pathological evidence to confirm the absence of appendicitis.

We found an ultrasound sensitivity and specificity of 81.4% and 91.43%, respectively. Likewise, Hwang found the sensitivity and specificity to be 86% and 94%, respectively [22]. In contrast, Giljaca et al. reported 69% sensitivity and 81% specificity [23]. Similar findings with a US accuracy of 77.5%, sensitivity of 80%, and specificity of 60% have been reported by Farooq et al. [24]. Alelyani et al. reported inconsistent US accuracy, sensitivity, and specificity trends that were 46.2%, 38.9% and 89.5%, respectively [25]. Despite fluctuations between the values of sensitivities and specificities, the specificity of US is markedly higher compared to the sensitivity across all studies, which plausibly indicates the technical limitation of US. Factors such as expertise or experience of the radiologist may also contribute to the statistically significant and increased sensitivity of CT compared to US [26]. Therefore, when there is a disagreement between scoring and the clinical evaluation, US should be preferred as it is less harmful, and CT should be performed when US is negative or inconclusive [27].

Our study found that the PPV and NPV of US for diagnosing appendicitis were 85.07% and 95.4%, respectively. Other studies, such as one by Hwang in 2018, reported slightly higher pooled values for PPV (100%) and NPV (92%) [22]. Debnath et al. in 2015 reported a PPV of 92.6% and an NPV of 71.6% for US [28], while Löfvenberg and Salö in 2016 reported PPV and NPV values of 92% and 93%, respectively [29]. The variation in PPV and NPV values across the studies depends on several factors outlined previously in this study.

Among other imaging techniques, US is still considered the first examination choice in suspected cases of appendicitis. In the absence of a definitive pathological finding, those patients presenting with signs of atypical appendicitis during a clinical examination (lack of at least one of the classic signs, including fever, wandering pain, pain on palpation of McBurney’s point or elevated inflammation values in lab tests) are suggested a further diagnostic workup. For such cases, especially male patients, CT (unenhanced low dose) or MRI (unenhanced) is suggested. Patients (men and women, excluding pregnant women) with laboratory parameters such as significantly deranged CRP values and suspected perforation are advised to go for CT with intravenous contrast agents to rule out appendicitis-associated pathologies [30]. Due to the high specificity and relatively low sensitivity of US, we corroborate its efficacy in diagnosing appendicitis across varied presentations (appendicitis, acute appendicitis, appendicitis with the absence or presence of secondary signs, and appendicitis with or without comorbidities).

Limitations

This study has several limitations that need to be considered. First, the study design was retrospective, which may impact the accuracy and reliability of the results. Additionally, the evaluation period was relatively short, and the sample size was relatively small, limiting the generalizability of the findings. Another limitation is the absence of body mass index data, which can be difficult to obtain using ultrasound imaging, particularly in obese patients. Furthermore, the sensitivity of ultrasound imaging can vary significantly and largely depends on the expertise of the radiologist interpreting the results. Last, our study's age range only included individuals aged 14 years and above, which limits its generalizability to the population under the 14-year age group.

## Conclusions

Since the diagnosis of appendicitis remains a serious concern, imaging techniques continue to be the best modality for accurate diagnosis and to provide the best patient care. Ultrasound is an effective choice of imaging for the initial diagnosis of appendicitis in male patients. However, a CT scan should be prescribed for further evaluation in cases with US-negative appendicitis exhibiting secondary signs or pathologies to rule out acute appendicitis.
